# A predictive model for early death in elderly patients with gastric cancer: A population-based study

**DOI:** 10.3389/fonc.2022.972639

**Published:** 2022-08-22

**Authors:** Wenwei Yang, Yuting Fang, Yaru Niu, Yongkun Sun

**Affiliations:** ^1^ Department of Medical Oncology, National Cancer Center/National Clinical Research Center for Cancer/Cancer Hospital, Chinese Academy of Medical Sciences and Peking Union Medical College, Beijing, China; ^2^ Department of Medical Oncology, National Cancer Center/National Clinical Research Center for Cancer/Hebei Cancer Hospital, Chinese Academy of Medical Sciences, Langfang, China

**Keywords:** gastric cancer (GC), early death, aged, surveillance, epidemiology and end results (SEER), nomogram

## Abstract

**Background:**

The mean age of gastric cancer (GC) patients has increased due to the aging society. Elderly GC patients with poor physical status tend to develop complications during the treatment courses, which cause early death. This study aimed to identify risk factors and establish nomograms for predicting total early death and cancer-specific early death in elderly GC patients.

**Methods:**

Data for elderly GC patients were extracted from the Surveillance, Epidemiology, and End Results (SEER) database. These patients were randomly assigned to a training cohort and a validation cohort. The univariate logistic regression model and backward stepwise logistic regression model were used to identify independent risk factors for early death. Nomograms were constructed to predict the overall risk of early death and their performance was validated by receiver operating characteristic (ROC) curve, calibration curve, decision curve analyses (DCA), integrated discrimination improvement (IDI), and net reclassification improvement (NRI) in both training and validation cohorts.

**Results:**

Among the 3102 enrolled patients, 1114 patients died within three months from the first diagnosis and 956 of them died due to cancer-specific causes. Non-Asian or Pacific Islander (API) race, non-cardia/fundus or lesser/greater curvature, higher AJCC stage, no surgery and no chemotherapy were all related to a high risk of both all-cause early death and cancer-specific early death. Higher T stage and N0 stage were only positively related to total early mortality, while liver metastasis was only positively related to cancer-specific early mortality. Based on these identified factors, two nomograms were developed for predicting the risk of all-cause and cancer-specific early death, which showed good performance with the AUC of the nomograms were 0.775 and 0.766, respectively. The calibration curves, DCAs, NRI, and IDI also confirmed the value of these nomograms.

**Conclusions:**

These nomogram models were considered a practical tool to identify the early death of elderly GC patients and help provide a more individualized treatment strategy.

## Introduction

Gastric cancer (GC) is thes fifth most commonly diagnosed cancer and the fourth leading cause of cancer-related mortality worldwide, with over one million new cases worldwide reported in 2020 (1). However, due to the unspecific symptoms and late symptom presentation, most GC patients are diagnosed at advanced stages with metastases (2). Almost one-third of gastric cancers were diagnosed at an advanced stage (3). Patients with advanced gastric cancer have a poor prognosis, with a low 5-year survival rate and a median survival time of around 13–16 months (4).

Although, in recent years, remarkable advances in the treatment of GC have notably improved the prognosis of GC patients, early death (die within three months of initial diagnosis) of GC patients is still an intractable problem (5, 6). Many factors might determine the prognosis of gastric cancer, such as age, sex, race, tumor location, tumor differentiation, TNM stage, surgery, chemotherapy, and radiation (7).

Currently, with the rapid aging of the global population, the average age of GC patients has gradually increased (8). The elderly GC patients always have poor physical or nutritional status, and they are more prone to developing a higher incidence of complications and mortality than non-elderly patients during the period of treatment, which finally leads to an early death (9). In this context, the efficacy and safety of treatment should be more carefully considered when deciding on treatment strategies for elderly patients with gastric cancer.

At present, clinicians mainly use the American Joint Committee on Cancer (AJCC) TNM staging system to evaluate the prognosis of gastric cancer patients (10). However, this assessment approach is inaccurate as many risk factors associated with prognosis were not considered in the TNM staging system and we cannot directly evaluate the prognosis of elderly GC patients by this system. Accordingly, it is essential to recognize the risk factors related to early mortality for elderly GC patients, and then develop a novel model to assess the prognosis of this group of patients accurately. This model may assist clinicians in promptly identifying the older GC patients at high risk of early death and scheduling individualized treatment strategies and supportive care for them, improving their survival and life quality.

Large-scale studies are urgently needed, while few specifically evaluated the prognosis of elderly patients with GC. Therefore, in this study, we used Surveillance, Epidemiology, and End Results (SEER) database to analyze the clinicopathological characteristics of elderly patients with GC and identify the predictors of early mortality. Furthermore, based on these risk factors, we established predictive models for the early death of elderly GC patients and validated the accuracy of the models.

## Methods

### Data source

A retrospective cohort study was conducted using data from the Surveillance, Epidemiology and End Results (SEER) database, containing cancer patients’ demographic, clinicopathological, and survival data. These data were collected from 18 established cancer registries covering about a third of the United States population and updated annually (11).

The database version used in this study was released in April 2019, named Incidence-SEER 18 Regs Custom Data (with additional treatment fields), Nov 2018 Sub (1975-2016 varying). SEER*Stat Software (www.seer.cancer.gov, version 8.4.0) was used to extract data about gastric cancer patients from the SEER database.

This study complied with all relevant ethical standards including the 1964 Declaration of Helsinki. As cancer is a reportable disease in the United States, the SEER database is a public-use database and the data released by the SEER database do not require informed patient consent.

### Patient selection

As the SEER database only collected information about organ metastases from 2010, the elderly GC patients diagnosed from 2010 to 2015 were included in this study. There is no standard definition of “elderly” exists; thus, we defined “elderly GC patients” as GC patients with age≥75 years old in this review (12, 13). The 3rd edition of International Classification of Diseases for Oncology (ICD‐O‐3) criteria was used to identify GC patients according to the primary site (C16.0–C16.6, C16.8–C16.9), and the diagnosis of these patients should also be histologically confirmed.

**Figure 1:**
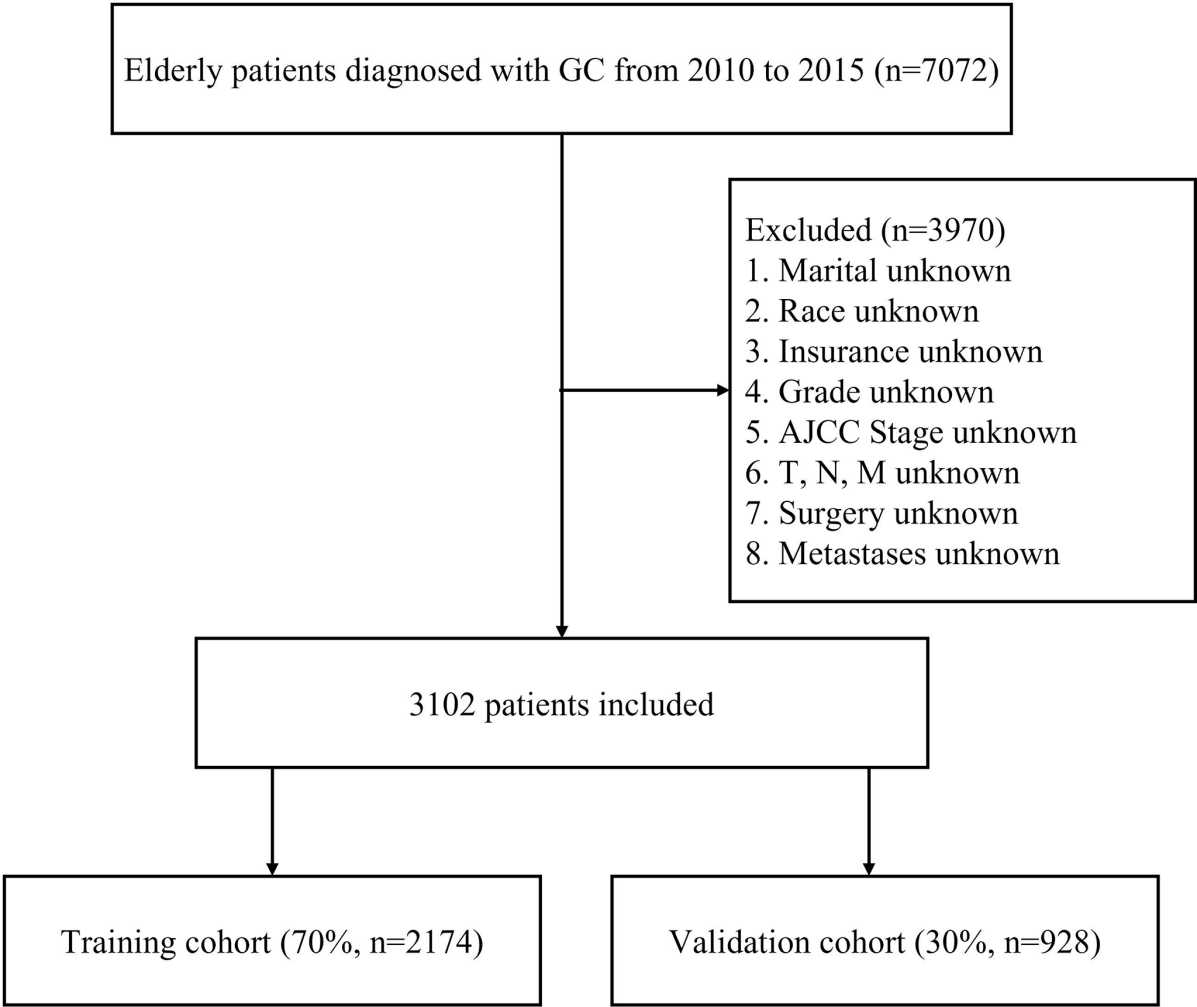
Flowchart of data selection of the elderly GC patients from the SEER database.

Patients who met any of the following criteria were excluded: diagnosed based on autopsy or the death certificate, diagnosed without the pathological diagnosis, multiple primary tumors, unknown race, stage Tis, T0, Tx, or NX, unknown information of distant metastases, incomplete follow-up or with unknown information on the extracted variables. This study defined early death as cancer-specific death within three months of initial diagnosis (14). Finally, 3102 elderly GC patients were included in the study and randomly divided into two cohorts (7:3): the training cohort (2174 patients) and the validation cohort (928 patients). The flowchart of patient selection process is summarized in **Figure 1**.

The following variables were extracted from the SEER database: age at diagnosis, year of diagnosis, race, sex, marital status, primary site, differentiation grade, 7th AJCC grade, T stage, N stage, M stage, histological grade, surgery status, radiotherapy status, chemotherapy status, insurance status, bone metastases, brain metastases, liver metastases, lung metastases, cause of death, and survival months.

### Statistical analysis

All included variables, except survival time, were described by the number and the percentage (N, %). Early death among elderly GC patients was stratified by metastatic site and number of metastatic organs. As mentioned above, these patients were randomly allocated into training and validation cohorts. The training cohort was used to establish a nomogram and the validation cohort was used to validate it. The baseline characteristics of the training and validation cohorts were displayed by the number and the percentage (N, %), and the *Pearson* chi-square test was used to explore the difference between these two cohorts.

Univariate logistic regression analyses, multivariate logistic regression analyses and the backward stepwise selection method were utilized to determine the prognostic factors related to the early death of elderly gastric cancer patients in the training cohort. Variables significantly associated with early death in the multivariate analyses were selected to develop nomograms. The predictive nomograms for all-cause early death and cancer-specific early death were developed, respectively.

To assess the reliability of the nomograms, the area under the time-dependent receiver operating characteristic (ROC) curve, calibration curves and decision curve analysis (DCA) were conducted in the training and validation cohort, respectively. The area under the ROC curve (AUC) was used to measure the discrimination ability of the nomogram. Calibration curves represented the agreement between the observed outcome and predicted probability by bootstrapping with 200 resamples. DCA was used to assess the value of the nomogram in clinical applications in all patients. To compare our models with the TNM staging system, integrated discrimination improvement (IDI) and net reclassification improvement (NRI) were calculated.

Statistical analyses were performed using the R software (version 4.1.2) and SPSS statistics software (version 17; IBM Corp., Armonk, NY, USA). A two-sided P value <0.05 was considered statistically significant.

## Results

### Demographic and clinical characteristics

Based on the inclusion and exclusion criteria, 3102 elderly patients (age ≥ 75 years old) diagnosed with GC from 2010 to 2015 in the SEER database were finally included in the study. Fifty-eight point one percent of patients (N=1802) were male and the majority of patients were Whites (71.2%, N=2208). Most of the patients were insured (99.3%, N=3080), while 50.0% of patients (N=1551) were unmarried. As for the tumor histology, 75.3% of patients had adenocarcinoma and 13.5% had signet ring cell carcinoma. Among the study population, the most frequent primary sites were cardia and gastric fundus (31.5%) and the second most frequent in antrum and pylorus (26.9%). The most common organ of metastasis was the liver (12.0%), followed by the lung (3.7%), bone (2.1%) and brain (0.2%). The demographic and clinicopathological characteristics of elderly GC patients are shown in **Table 1**.

**Table 1:** Demographic and clinicopathological characteristics of elderly GC patients with or without early death.

Characteristics	Number of patients (%)			
	Overall n=3102	No early death n=1988	All-cause early death n=1114	Cancer-specific early death n=956
**Insurance status**				
Insured	3080 (99.3)	1976 (99.4)	1104 (99.1)	947 (99.1)
Uninsured	22 (0.7)	12 (0.6)	10 (0.9)	9 (0.9)
**Marital status**				
Married	1551 (50.0)	1042 (52.4)	509 (45.7)	447 (46.8)
Unmarried	1551 (50.0)	946 (47.6)	605 (54.3)	509 (53.2)
**Race**				
White	2208 (71.2)	1395 (70.2)	813 (73.0)	696 (72.8)
Black	346 (11.2)	204 (10.3)	142 (12.7)	126 (13.2)
Other	548 (17.7)	389 (19.6)	159 (14.3)	134 (14.0)
**Sex**				
Male	1802 (58.1)	1161 (58.4)	641 (57.5)	551 (57.6)
Female	1300 (41.9)	827 (41.6)	473 (42.5)	405 (42.4)
**Primary site**				
Cardia/fundus	976 (31.5)	675 (34.0)	301 (27.0)	266 (27.8)
* Body*	297 (9.6)	180 (9.1)	117 (10.5)	97 (10.1)
Antrum/pylorus	835 (26.9)	518 (26.1)	317 (28.5)	267 (27.9)
Lesser/greater curvature	383 (12.3)	272 (13.7)	111 (10.0)	92 (9.6)
Other	611 (19.7)	343 (17.3)	268 (24.1)	234 (24.5)
**Differentiation grade**				
I	169 (5.4)	128 (6.4)	41 (3.7)	33 (3.5)
II	879 (28.3)	598 (30.1)	281 (25.2)	230 (24.1)
III	1984 (64.0)	1221 (61.4)	763 (68.5)	670 (70.1)
IV	70 (2.3)	41 (2.1)	29 (2.6)	23 (2.4)
**Histology**				
Adenocarcinoma	2337 (75.3)	1519 (76.4)	818 (73.4)	703 (73.5)
Signet ring cell carcinoma	418 (13.5)	259 (13.0)	159 (14.3)	140 (14.6)
Other	347 (11.2)	210 (10.6)	137 (12.3)	113 (11.8)
**AJCC Stage**				
I	847 (27.3)	540 (27.2)	307 (27.6)	246 (25.7)
II	576 (18.6)	440 (22.1)	136 (12.2)	101 (10.6)
III	870 (28.0)	624 (31.4)	246 (22.1)	215 (22.5)
IV	809 (26.1)	384 (19.3)	425 (38.2)	394 (41.2)
**T stage**				
T1	1003 (32.3)	569 (28.6)	434 (39.0)	365 (38.2)
T2	363 (11.7)	258 (13.0)	105 (9.4)	89 (9.3)
T3	929 (29.9)	682 (34.3)	247 (22.2)	206 (21.5)
T4	807 (26.0)	479 (24.1)	328 (29.4)	296 (31.0)
**N stage**				
N0	1573 (50.7)	942 (47.4)	631 (56.6)	530 (55.4)
N1	816 (26.3)	524 (26.4)	292 (26.2)	262 (27.4)
N2	320 (10.3)	247 (12.4)	73 (6.6)	64 (6.7)
N3	393 (12.7)	275 (13.8)	118 (10.6)	100 (10.5)
**M stage**				
M0	2293 (73.9)	1604 (80.7)	689 (61.8)	562 (58.8)
M1	809 (26.1)	384 (19.3)	425 (38.2)	394 (41.2)
**Surgery**				
No	1740 (56.1)	966 (48.6)	774 (69.5)	698 (73.0)
Yes	1362 (43.9)	1022 (51.4)	340 (30.5)	258 (27.0)
**Radiation**				
No/Unknown	2361 (76.1)	1363 (68.6)	998 (89.6)	851 (89.0)
Yes	741 (23.9)	625 (31.4)	116 (10.4)	105 (11.0)
**Chemotherapy**				
No/Unknown	2052 (66.2)	1093 (55.0)	959 (86.1)	813 (85.0)
Yes	1050 (33.8)	895 (45.0)	155 (13.9)	143 (15.0)
**Bone metastases**				
No	3038 (97.9)	1957 (98.4)	1081 (97.0)	926 (96.9)
Yes	64 (2.1)	31 (1.6)	33 (3.0)	30 (3.1)
**Brain metastases**				
No	3095 (99.8)	1986 (99.9)	1109 (99.6)	951 (99.5)
Yes	7 (0.2)	2 (0.1)	5 (0.4)	5 (0.5)
**Liver metastases**				
No	2731 (88.0)	1828 (92.0)	903 (81.1)	753 (78.8)
Yes	371 (12.0)	160 (8.0)	211 (18.9)	203 (21.2)
**Lung metastases**				
No	2987 (96.3)	1942 (97.7)	1045 (93.8)	893 (93.4)
Yes	115 (3.7)	46 (2.3)	69 (6.2)	63 (6.6)

### Incidence of early death

**Figure 2:**
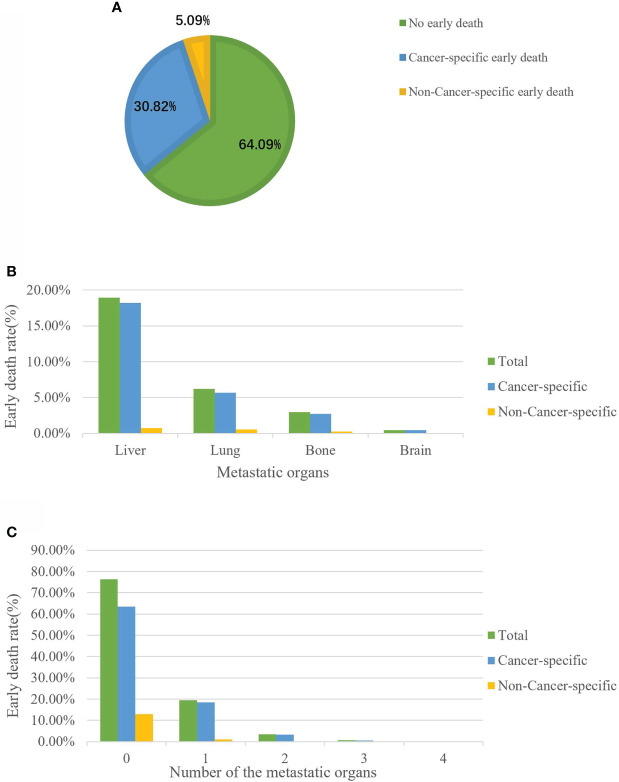
The incidence of cancer-specific early death among elderly GC patients **(A)**. Distribution of early death among elderly GC patients stratified by metastatic site **(B)** and the number of metastatic organs **(C)**.

Among the 3102 elderly GC patients, 1114 (35.91%) experienced all-cause early death. Of these early death patients, 956 patients died from cancer-specific causes and 158 died from non-cancer-specific causes (**Figure 2A**). Among all early death patients, the most common metastatic organ was liver (18.94%), followed by lung (6.19%), bone (2.96%) and brain (0.45%). Moreover, patients with brain metastasis all died from cancer-related causes (**Figure 2B**). We also described the early death rate of patients with different numbers of metastatic organs (**Figure 2C**).

### Identification of early death prognostic factors

Based on random assignment, 2174 patients were allocated into the training cohort and 928 into the validation cohort. In both the training and validation cohorts, most of the patients were male (58.7% and 56.6%), Whites (70.9% and 71.9%), and insured (99.4% and 99.0%). The proportions of married and unmarried patients were approximately the same in both training cohort (49.8% vs. 50.2%) and validation cohort (50.4% vs. 49.6%). The most frequent primary sites were cardia and gastric fundus (31.9% and 30.5%), and the most common histological type was adenocarcinoma (75.9% and 74.0%). The most frequently reported differentiation grade was poor differentiation (64.0% and 63.9%). Patients at stage T1 (32.8% and 31.2%), stage N0 (51.0% and 50.1%), and stage M0 (72.9% and 76.4%) were the most common. As for treatment, fewer of the patients underwent surgery (43.7% and 44.4%), radiotherapy (23.6% and 24.7%) and chemotherapy (33.3% and 35.0%). Liver was the most common metastatic site displayed in both cohorts (12.9% and 9.8%). Except for the AJCC stage, M stage and liver metastases, all other variables were distributed similarly in the two cohorts (p>0.05). More detailed baseline characteristics of these two cohorts are given in **Table 2**.

**Table 2:** The baseline characteristics of the training and validation cohorts.

Characteristics	Number of patients (%)		P value
	Training cohort (n=2174)	Validation cohort (n=928)
**Insurance status**			
Insured	2161 (99.4)	919 (99.0)	0.37
Uninsured	13 (0.6)	9 (1.0)	
**Marital status**			
Married	1083 (49.8)	468 (50.4)	0.784
Unmarried	1091 (50.2)	460 (49.6)	
**Race**			
White	1541 (70.9)	667 (71.9)	0.856
Black	245 (11.3)	101 (10.9)	
Other	388 (17.8)	160 (17.2)	
**Sex**			
Male	1277 (58.7)	525 (56.6)	0.28
Female	897 (41.3)	403 (43.4)	
**Primary site**			
Cardia/fundus	693 (31.9)	283 (30.5)	0.776
Body	211 (9.7)	86 (9.3)	
Antrum/pylorus	589 (27.1)	246 (26.5)	
Lesser/greater curvature	262 (12.1)	121 (13.0)	
Other	419 (19.3)	192 (20.7)	
**Differentiation grade**			
I	123 (5.7)	46 (5.0)	0.507
II	616 (28.3)	263 (28.3)	
III	1391 (64.0)	593 (63.9)	
IV	44 (2.0)	26 (2.8)	
**Histology**			
Adenocarcinoma	1650 (75.9)	687 (74.0)	0.524
Signet ring cell carcinoma	288 (13.2)	130 (14.0)	
Other	236 (10.9)	111 (12.0)	
**AJCC Stage**			
I	606 (27.9)	241 (26.0)	0.04
II	393 (18.1)	183 (19.7)	
III	585 (26.9)	285 (30.7)	
IV	590 (27.1)	219 (23.6)	
**T stage**			
T1	713 (32.8)	290 (31.2)	0.713
T2	257 (11.8)	106 (11.4)	
T3	650 (29.9)	279 (30.1)	
T4	554 (25.5)	253 (27.3)	
**N stage**			
N0	1108 (51.0)	465 (50.1)	0.712
N1	563 (25.9)	253 (27.3)	
N2	231 (10.6)	89 (9.6)	
N3	272 (12.5)	121 (13.0)	
**M stage**			
M0	1584 (72.9)	709 (76.4)	0.044
M1	590 (27.1)	219 (23.6)	
**Surgery**			
No	1224 (56.3)	516 (55.6)	0.749
Yes	950 (43.7)	412 (44.4)	
**Radiation**			
No/Unknown	1662 (76.4)	699 (75.3)	0.53
Yes	512 (23.6)	229 (24.7)	
**Chemotherapy**			
No/Unknown	1449 (66.7)	603 (65.0)	0.39
Yes	725 (33.3)	325 (35.0)	
**Bone metastases**			
No	2129 (97.9)	909 (98.0)	1
Yes	45 (2.1)	19 (2.0)	
**Brain metastases**			
No	2167 (99.7)	928 (100.0)	0.188
Yes	7 (0.3)	0 (0.0)	
**Liver metastases**			
No	1894 (87.1)	837 (90.2)	0.019
Yes	280 (12.9)	91 (9.8)	
**Lung metastases**			
No	2087 (96.0)	900 (97.0)	0.22
Yes	87 (4.0)	28 (3.0)	

Univariate logistic regression analyses for the training cohort displayed insurance status, race, primary site, differentiation grade, histologic type, AJCC stage, T stage, N stage, M stage, bone metastases, lung metastases and liver metastases were all associated with all-cause and cancer-specific early death. Moreover, marital status and brain metastases were also related to cancer-specific early death (**Table 3**).

**Table 3:** Univariate logistic regression for analyzing the prognostic factors for early death.

Variable	All-cause early death			Cancer-specific early death		
	OR	95%CI	P value	OR	95%CI	P value
**Sex**						
Male	1(ref)			1(ref)		
Female	1.49	0.50-4.44	0.477	1.9	0.63-5.66	0.252
**Insurance status**						
Insured	1(ref)			1(ref)		
Uninsured	1.36	1.14-1.62	0.001	1.24	1.04-1.49	0.02
**Marital status**						
Married	1(ref)			1(ref)		
Unmarried	1.19	0.90-1.56	0.217	1.32	1.00-1.75	0.049
**Race**						
White	1(ref)			1(ref)		
Black	0.71	0.56-0.90	0.005	0.74	0.58-0.95	0.02
Other	1.12	0.94-1.33	0.22	1.1	0.92-1.32	0.308
**Primary site**						
Cardia/fundus	1(ref)			1(ref)		
Body	1.38	1.00-1.90	0.052	1.25	0.89-1.75	0.193
Antrum/pylorus	1.57	1.25-1.98	<0.001	1.39	1.09-1.77	0.007
Lesser/greater curvature	0.85	0.62-1.17	0.333	0.82	0.58-1.14	0.231
Other	2.04	1.59-2.62	<0.001	1.87	1.45-2.43	<0.001
**Differentiation grade**						
I	1(ref)			1(ref)		
II	1.31	0.85-2.01	0.223	1.43	0.90-2.29	0.131
III	1.65	1.1-2.49	0.016	1.89	1.21-2.95	0.005
IV	1.99	0.97-4.07	0.06	1.93	0.90-4.12	0.09
**Radiation**						
No/Unknown	1(ref)			1(ref)		
Yes	1.06	0.82-1.37	0.663	1.08	0.83-1.41	0.568
**Histology**						
Adenocarcinoma	1(ref)			1(ref)		
Signet ring cell	1.27	0.96-1.68	0.089	1.18	0.89-1.58	0.249
Other	0.55	0.41-0.74	<0.001	0.49	0.35-0.67	<0.001
**AJCC Stage**						
I	1(ref)			1(ref)		
II	0.79	0.62-1.00	0.055	0.87	0.67-1.12	0.289
III	2.1	1.66-2.65	<0.001	2.44	1.92-3.09	<0.001
IV	0.52	0.38-0.71	<0.001	0.53	0.38-0.74	<0.001
**T stage**						
T1	1(ref)			1(ref)		
T2	0.53	0.42-0.67	<0.001	0.54	0.43-0.69	<0.001
T3	1	0.80-1.25	0.989	1.11	0.88-1.39	0.387
T4	0.77	0.62-0.95	0.013	0.83	0.67-1.04	0.1
**N stage**						
N0	1(ref)			1(ref)		
N1	0.52	0.38-0.71	<0.001	0.55	0.39-0.77	<0.001
N2	0.68	0.51-0.90	0.007	0.68	0.51-0.92	0.011
N3	2.63	2.16-3.19	<0.001	3	2.46-3.66	<0.001
**M stage**						
M0	1(ref)			1(ref)		
M1	0.45	0.38-0.54	<0.001	0.36	0.30-0.44	<0.001
**Surgery**						
No	1(ref)			1(ref)		
Yes	0.23	0.18-0.30	<0.001	0.27	0.21-0.36	<0.001
**Chemotherapy**						
No/Unknown	1(ref)			1(ref)		
Yes	0.2	0.16-0.25	<0.001	0.25	0.20-0.31	<0.001
**Bone metastases**						
No	1(ref)			1(ref)		
Yes	1.83	1.02-3.31	0.044	1.96	1.08-3.54	0.027
**Brain metastases**						
No	1(ref)			1(ref)		
Yes	4.35	0.84-22.47	0.079	5.54	1.07-28.62	0.041
**Liver metastases**						
No	1(ref)			1(ref)		
Yes	2.64	2.04-3.40	<0.001	3.13	2.43-4.05	<0.001
**Lung metastases**						
No	1(ref)			1(ref)		
Yes	2.68	1.73-4.15	<0.001	2.71	1.76-4.17	<0.001

OR, odds ratio; ref, reference.

All these significant risk factors identified in the univariate logistic regression analyses were incorporated into the multivariate logistic analyses and backward stepwise analyses. The results revealed that race, primary site, AJCC stage, surgery and chemotherapy were independent prognostic factors for both all-cause and cancer-specific early death of elderly GC patients (**Table 4**). T stage and N stage were only significantly related to all-cause early death, while liver metastasis was only significantly associated with cancer-specific early death.

**Table 4:** Multivariate logistic regression for analyzing the risk factors for early death.

Variable	All-cause early death			Cancer-specific early death		
	OR	95%CI	P value	OR	95%CI	P value
**Race**						
White	1(ref)			1(ref)		
Black	0.96	0.70-1.32	0.796	1.11	0.80-1.54	0.523
Other	0.59	0.44-0.77	<0.001	0.65	0.49-0.87	0.003
**Primary site**						
Cardia/fundus	1(ref)			1(ref)		
Body	1.31	0.90-1.91	0.152	1.22	0.83-1.80	0.308
Antrum/pylorus	1.5	1.13-2.00	0.005	1.34	1.00-1.79	0.053
Lesser/greater curvature	0.94	0.65-1.35	0.72	0.92	0.63-1.35	0.67
Other	1.71	1.27-2.31	<0.001	1.55	1.14-2.10	0.005
**Differentiation grade**						
I	1(ref)			1(ref)		
II	1.13	0.70-1.81	0.62	1.14	0.68-1.92	0.611
III	1.54	0.97-2.44	0.069	1.63	0.99-2.68	0.056
IV	1.74	0.78-3.89	0.176	1.58	0.68-3.69	0.292
**AJCC Stage**						
I	1(ref)			1(ref)		
II	1.07	0.70-1.63	0.758	0.75	0.49-1.17	0.203
III	1.96	1.21-3.17	0.006	1.39	0.90-2.15	0.133
IV	3.62	2.50-5.22	<0.001	2.39	1.62-3.53	<0.001
**T stage**						
T1	1(ref)			1(ref)		
T2	0.69	0.48-0.99	0.045	0.78	0.53-1.15	0.205
T3	0.87	0.60-1.25	0.444	1.03	0.71-1.49	0.895
T4	1.07	0.73-1.57	0.729	1.41	0.96-2.07	0.083
**N stage**						
N0	1(ref)			1(ref)		
N1	0.76	0.57-1.01	0.055	NA	NA	NA
N2	0.55	0.36-0.84	0.006	NA	NA	NA
N3	0.69	0.46-1.04	0.08	NA	NA	NA
**Surgery**						
No	1(ref)			1(ref)		
Yes	0.41	0.32-0.53	<0.001	0.3	0.23-0.39	<0.001
**Chemotherapy**						
No/Unknown	1(ref)			1(ref)		
Yes	0.14	0.11-0.18	<0.001	0.16	0.13-0.21	<0.001
**Liver metastases**						
No	1(ref)			1(ref)		
Yes	NA	NA	NA	1.48	1.02-2.14	0.039

OR, odds ratio; ref, reference. NA, not applicable.

Furthermore, elderly GC patients with a primary site at antrum and pylorus, and AJCC stage III and IV were more likely to die within three months for all causes, while patients who had tumors at T2 stage, N2 stage, and had done surgery or chemotherapy with race except Blacks and Whites were less likely to experience all-cause early death. For cancer-specific early death, patients whose tumor occurred at the site except for cardia, fundus, body, antrum, pylorus, and lesser or greater curvature, and with AJCC stage III and liver metastases were more easily to experience early mortality. Patients who had done surgery or chemotherapy with race except Blacks and Whites were less likely to die early. More detailed information is shown in **Table 4**.

### Nomogram construction

**Figure 3:**
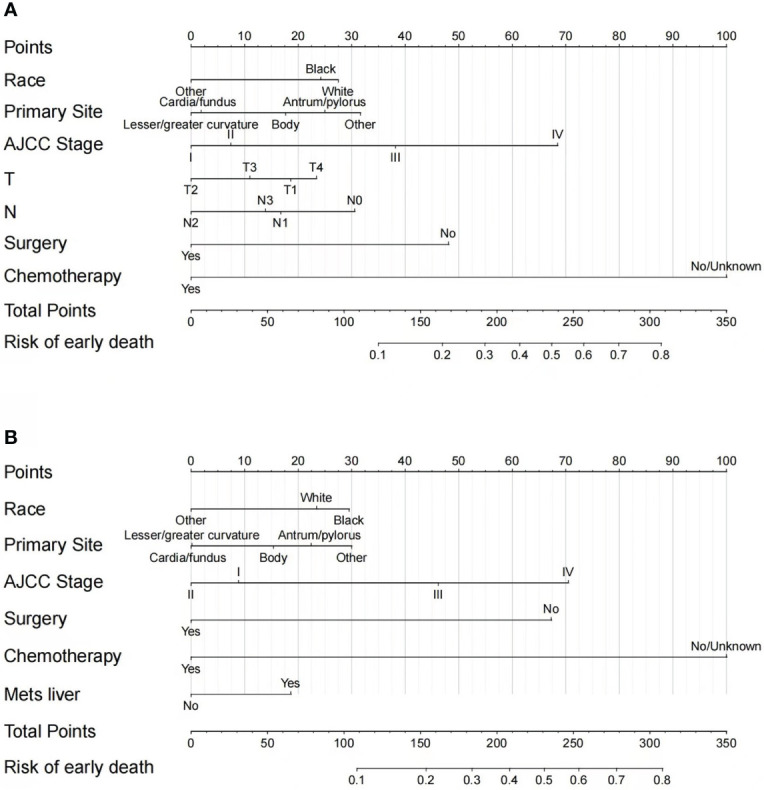
Nomograms for predicting all-cause **(A)** and cancer-specific early death **(B)** in elderly GC patients.

According to the independent risk factors identified from multivariate logistic regression, two nomograms were constructed to predict the risk of all-cause early death (**Figure 3A**) and cancer-specific early death (**Figure 3B**) among elderly GC patients, respectively. Each prognostic factor had its corresponding points, which converted the risk of each factor to a calculable value. The total number of points could be calculated by adding the points for all prognostic factors up, corresponding to the probability of early death by drawing a vertical line to the bottom probability line. The probability of all-cause early death and cancer-specific early death ranged from 0.1 to 0.80. Therefore, not every total number of points would have a corresponding probability. From the nomograms, we can see that chemotherapy had the strongest prognostic value in predicting early mortality.

### Nomogram assessment

**Figure 4:**
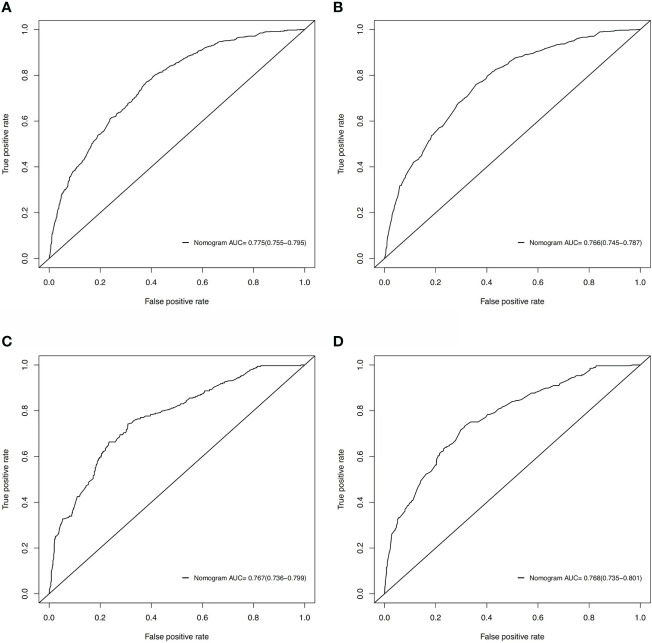
ROC curves for discrimination of the nomograms in predicting all-cause and cancer-specific early death in the training cohort **(A, B)** and the validation cohort **(C, D)**.

**Figure 5:**
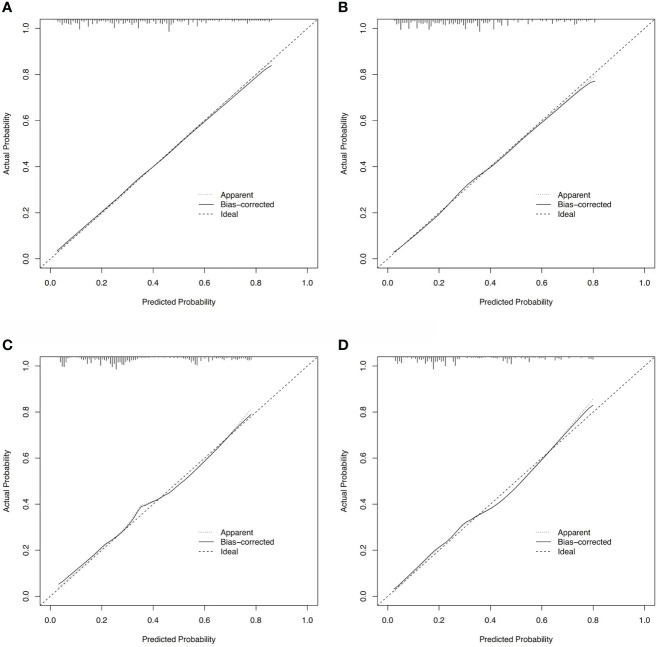
Calibration curves for assessing the calibration of the nomogram in predicting all-cause and cancer-specific early death in the training cohort **(A, B)** and the validation cohort **(C, D)**.

**Figure 6:**
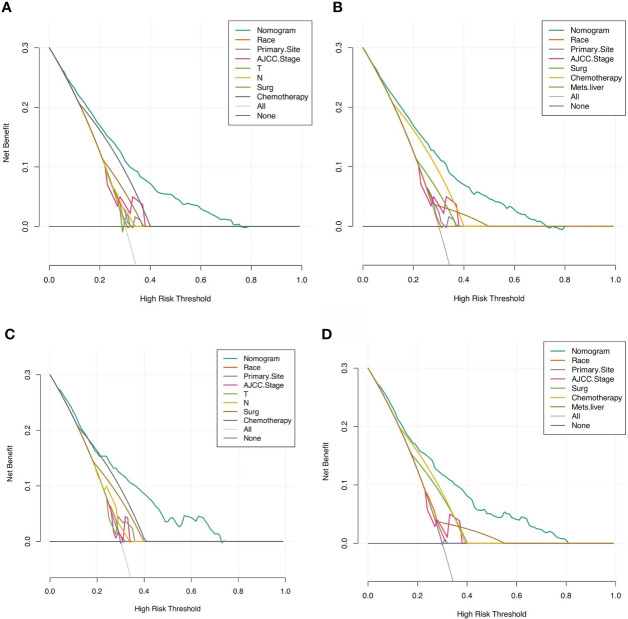
DCAs for the nomograms in predicting all-cause and cancer-specific early death in the training cohort **(A, B)** and the validation cohort **(C, D)**.

Based on these ROC curves, in the training cohort, the AUC for the nomograms to predict the risk of all-cause early death and cancer-specific early death were 0.775 and 0.766 (**Figures 4A, B**), respectively. The AUC of the nomograms for all-cause early death and cancer-specific early death prediction in the validation cohort were 0.767 and 0.768, respectively (**Figures 4C, D**). All nomograms exhibited satisfactory discrimination ability. The calibration curves for the two nomograms were close to the diagonal line in the training (**Figures 5A, B**) and validation cohort (**Figures 5C, D**), indicating great agreement between observed outcome and predictions. In addition, DCA graphically showed ideal net benefits of the nomograms for predicting all-cause and cancer-specific early death in both training cohort (**Figures 6A, B**) and validation cohort (**Figures 6C, D**), which suggested these nomograms had good clinical utility and this combined effect was better than that of a single variable. The results of the assessments all demonstrated the superiority of our prognostic nomograms.

The nomograms were also compared with the 7^th^ AJCC TNM staging system. For overall early death, the NRI values of the nomogram were 0.4379 (95%CI: 0.3763–0.4994) and 0.4257 (95% CI: 0.327–0.5245) in the training and validation cohorts, respectively. And the corresponding IDI values were 0.1444(95% CI: 0.1296–0.1593) and 0.1555(95%CI: 0.1315–0.1794). The NRI values of the nomogram for cancer-specific early death were 0.4058 (95%CI: 0.3408–0.4709) and 0.422 (95% CI: 0.3132–0.5308) in the training and validation cohorts, respectively. The corresponding IDI values of the nomogram for cancer-specific early death were 0.1266 (95% CI: 0.1121–0.1411) and 0.1463 (95%CI: 0.1228–0.1697). These findings indicated that our models had better recognition ability and were superior to other commonly used staging systems.

## Discussion

Even though the constant development of gastric cancer treatments, including targeted drugs, immunotherapy, and optimized surgical approach, has prolonged the survival time, GC’s morbidity and mortality rates are still high (1, 15, 16). Moreover, a retrospective study found that the early mortality rate remained stable from 2010 to 2015 (17). However, few studies have assessed the early death of gastric cancer. Furthermore, with population aging, the ages of GC patients have increased significantly. Elderly patients with poor physical status and comorbidities are more likely to experience early death than younger patients. Therefore, it is necessary to pay more attention to the early death of elderly GC patients and identify prognostic factors related to the early death of this group of patients, which helps to recognize the elderly GC patients who are more prone to die early and determines the treatment strategy for elderly GC patients specifically. Currently, many studies investigated the early death of patients with metastatic GC, but there is no study about the early death of elderly GC patients (17–19). To our knowledge, this is the first study to explore prognostic factors and construct nomograms for recognizing the early death of elderly patients with gastric cancer.

In the present study, 35.91% of elderly GC patients experienced death within three months after the initial diagnosis. Among these all-cause early deaths, 85.82% succumbed to cancer and 14.18% were not cancer-specific. However, another previous study showed that among 393 dead GC patients, 65.9% died due to cancer, while 34.1% of cases were not cancer-specific (20). The significant increase in the proportion of cancer-specific death in our study indicated that the elderly GC patients who tend to die early were more likely to die from cancer. This phenomenon suggests that even the older adults might be frail, they still need to receive timely tumor therapy if they can tolerate the tumor treatment. However, this finding was opposite to a previous hypothesis that old patients with multiple morbidities and poor performance status were likely to experience dysfunction of multiple organs, which consequently increased the death from non-cancer diseases. Therefore, our results need to be interpreted cautiously, and further large-scale studies are warranted. Moreover, we found that patients with brain metastasis all died from cancer-related causes in this study. However, GC with brain metastases is extremely rare (less than 1%) and the sample size is relatively small (21); thus, the positive relation between brain metastasis with cancer-specific early death cannot be inferred persuasively. Our results need more large-scale prospective studies to verify.

In our study, several variables were found to be independent risk factors related to all-cause early death and cancer-specific early death in elderly GC patients. Regarding overall early mortality, non-Asian or Pacific Islander (API) race, non-cardia/fundus or lesser/greater curvature, higher AJCC stage, higher T stage, and N0 stage were all associated with a high risk of all-cause early death. Surgery and chemotherapy were both negatively related to early death. When analyzing the cancer-specific early mortality, we found that non-API race, non-cardia/fundus or lesser/greater curvature, higher AJCC stage (stage III or IV), and liver metastasis were positively related to early death. Surgery and chemotherapy were both negatively related to early death.

As for the treatment of gastric cancer, chemotherapy and surgery were both prognostic factors that could affect the early death of elderly GC patients. Previously, some retrospective studies reported that elderly GC patients had a significantly higher incidence of postoperative complications than young patients (9, 22), which might cause chemotherapy delay and affect the overall survival benefit for elderly GC patients. In contrast, Hashimoto et al. found that the incidence of complications did not differ significantly between old and young GC patients (14), which could be explained by advances in surgical technique, improved perioperative management, and the less invasive surgical approaches applied to elderly patients. This study suggested that gastrectomy was feasible in elderly GC patients. Consistently, our results indicated that patients who underwent surgery had a considerably lower risk of early death.

Even though the benefit of surgery is evident, several conditions should be altered before performing surgery and some measurements could be taken to improve the survival benefit for elderly GC patients. Firstly, pneumonia was considered the postoperative complication with a higher incidence in the elderly group (23), and it can be prevented by preoperative respiratory status assessment and perioperative respiratory interventions to improve surgery safety (24). Secondly, multiple preexisting comorbidities and poor nutritional status led to frailty, consequently increasing the likelihood of death. Therefore, perioperative nutritional support may be essential to improve the prognosis of old GC patients. Furthermore, we should avoid performing aggressive surgery on elderly GC patients with multiple comorbidities and poor nutritional status (25). Thirdly, preoperative screening may reduce the possibility of death from other undiagnosed malignancies. More large-scale prospective studies are needed to confirm the above opinions in the future.

According to our results, chemotherapy was another strong treatment-related prognostic predictor, which suggested that chemotherapy could bring survival benefits and should be administrated if patients could tolerate it. In addition, old patients with worse basic physical conditions and more comorbidities are less likely to tolerate the side effects of cytotoxic therapy; thus, choosing an optimal chemotherapy regimen and dosage are vital for elderly GC patients. Besides chemotherapy, with the development of precision medicine, targeted therapy and immunotherapy have achieved breakthroughs for gastric cancer with a good safety profile and better compliance and efficacy, finally improving the prognosis of elderly GC patients.

Race is a confirmed prognostic factor for GC patients and several studies found that API races had a lower risk of death than other races (26, 27). We also found that API patients had a lower risk of early death in elderly GC patients. Previous studies showed that, compared with non-API races, API races had higher rates of regular screening and aggressive treatment (e.g., surgery and radiotherapy) with a more positive attitude toward cancer treatment (28–30), which might explain the reasons why API patients had a lower risk of early death.

The primary site of tumors affected the prognosis as well. We found that old GC patients whose tumors occurred at the cardia, fundus, lesser or greater curvature had a lower risk of early death. Conversely, several published pieces of research summarized that patients with cardia GC had a worse prognosis than patients with non-cardia GC, because cardia GC was assumed to have aggressive biological behavior and was more likely to experience lymph node metastasis and recurrence (31–33). We investigated published study results to explain these contradictory results. As we have mentioned before, cardia GC was more prone to experiencing lymph node metastasis; thus, this group of patients would be more likely to undergo lymphadenectomy and adjuvant treatment. Amini et al. found that the 5-year survival rates of cardia GC patients who underwent R0 resection were no lower than those with non-cardia GC (32). Additionally, the relatively small sample size in our study might affect the accuracy of these findings.

AJCC stage system is the conventional model for predicting the prognosis of cancer patients, including T stage, N stage, and M stage. Our study confirmed the value of the AJCC stage system in predicting prognosis and found that the T stage and N stage were both independent risk factors for the early death of elderly GC patients. T stage reflects the tumor size and has been confirmed as an independent prognostic factor in several kinds of tumors (34–36). It could be measured by surgical resection or imaging and used as an index guiding whether surgery is needed. Large tumor size is usually associated with a poor prognosis. However, in our study, T4 stage was related to the highest risk for all-cause early death, followed by T1, T3 and T2 stages. This result differed from the previous opinion, and only existed in the all-cause early death group but not in the cancer-specific early death group. Similarly, lower N stage (N0 or N1) was also the independent risk factor for all-cause early death, but not for cancer-specific early death. On the one hand, we speculated that the GC patients with T1 stage and lower N stage were more likely to undergo surgery and prone to experiencing postoperative complications, leading to the early death of elderly GC patients. However, according to our data, the surgery rate for T1, T2, T3, and T4 stages were 22.53%, 43.25%, 53.28% and 59.98%, and that for N0, N1, N2, and N3 stages were 32.29%, 33.09%, 70.63%, and 91.09%, respectively. These results do not support this speculation. On the other hand, the elderly GC patients diagnosed with lower T or N stage without surgery usually have multiple comorbidities and poor performance status. This group of patients cannot tolerate surgery and tend to die of non-cancer causes.

Liver metastasis was related to unfavorable prognoses in gastric cancer patients, because it can cause organ system damage and tumor load increasing. Based on previous studies, the median survival time for GC patients with liver metastases was about 3 months and the 5-year survival rate was only 0–10% (37, 38). Consistently, our study confirmed that liver metastasis was an independent predictor for cancer-specific early death in old GC patients.

Several similar studies have been published in recent years. Zhang et al. developed the nomograms for elderly gastric cancer patients after gastrectomy both in overall and cancer-specific survival. After univariate analysis and multivariate analysis, sex, age, race, histological grade, TNM stage, lung metastasis and tumor size were used to establish nomogram in OS, while age, grade, TNM, and tumor size were included to construct the nomogram in CSS. These results are different from our study. Firstly, the study conducted by Zhang et al. developed the nomograms for elderly gastric cancer patients after gastrectomy, while we established the models for early death in elderly GC patients. Secondly, we conducted the stepwise regression to eliminate variables with multicollinearity, while they did not conduct such analysis to eliminate the multicollinearity between variables. We believe our study design is more rational than others, and our predictive models are more convincing (39). The pathologic type might affect the prognosis of GC patients. A study carried out by Chen et al. found that age, race, tumor size, TNM staging, total gastrectomy, and radiotherapy affected the prognosis of patients with signet-ring cell carcinoma and made a nomogram for them (40). Another study also built a nomogram model for resectable GC to individually predict survival benefits, and the nomogram illustrated that T stage, N stage, comprehensive treatment, age at diagnosis, histological grade, and tumor size contribute to the prognosis of resectable GC patients (41).

Currently, nomograms are widely used as prognostic models that help generate an individual probability of a clinical event by combining several prognostic variables, and they can aid clinical decision-making and contribute to optimizing cancer treatment towards personalized medicine (42). Based on the application of high-quality and large-sample data from the SEER database, the nomograms we developed incorporated more significant prognostic factors for the early death of elderly GC patients and these factors were all easily obtained from medical records. Compared with the existing prognostic model (i.e., AJCC staging system), the validation parameters, including the AUC, calibration curve, DCA, IDI, and NRI, demonstrated greater reliability and clinical validity of our predictive models, because our nomograms combined more prognostic factors.

As the prognostic variables might change during the treatment course, these nomograms can be conveniently used to predict the real-time risk of early mortality in elderly GC patients, including the patients not receiving any treatment and patients who had received treatment. They can assist clinicians in determining individualized treatment options, designing clinical studies, and adjusting the follow-up treatments. In addition, these nomograms can better guide communication and informed consent discussions with patients and their family members.

However, several limitations to the present study should be considered. Firstly, more potentially prognostic factors related to early death were not analyzed, such as the Eastern Cooperative Oncology Group performance score, background diseases, peritoneal metastasis, helicobacter pylori and sarcopenia, which might affect the predictive accuracy of the nomograms. Secondly, selection bias might exist because several cases were excluded due to missing data. Thirdly, our nomograms were only validated by an internal validation, and further external validation by more large prospective studies is necessary.

## Conclusion

As the world’s population ages, approximately one-third of elderly GC patients experience an early death. Several prognostic factors were found to be the independent risk factors associated with the all-cause and cancer-specific early death of elderly GC patients. Based on these factors, two predictive nomograms were developed and validated to predict the risk of all-cause early death and cancer-specific early death, respectively. The good performance of nomograms suggested that these models may assist clinicians in identifying elderly patients with a high risk of early death and providing personalized treatments for them, consequently improving their survival benefits. They also provide insights into the clinical judgment and design of clinical trials.

## Data availability statement

Publicly available datasets were analyzed in this study. This data can be found here: SEER database: Incidence-SEER 18 Regs Custom Data.

## Ethics statement

The studies involving human participants were reviewed and approved by SEER database, which is a public-use database. Written informed consent to participate in this study was provided by the participants’ legal guardian/next of kin.

## Author contributions

WY contributed to the study design and the composition of the manuscript and literature review. WY collected and analyzed the data. YF and YN helped to perform and check the statistical analysis. YS contributed to the supervision and supported final approval of the article. All authors contributed to manuscript revision, read and approved the final version.

## Funding

This work was supported by The National Key Research and Development Program of China (Grant No.2021YFF1201300).

## Conflict of interest

The authors declare that the research was conducted in the absence of any commercial or financial relationships that could be construed as a potential conflict of interest.

## Publisher’s note

All claims expressed in this article are solely those of the authors and do not necessarily represent those of their affiliated organizations, or those of the publisher, the editors and the reviewers. Any product that may be evaluated in this article, or claim that may be made by its manufacturer, is not guaranteed or endorsed by the publisher.
